# Transcription factor ZEB2 is essential for ureteral smooth muscle cell differentiation

**DOI:** 10.1371/journal.pgen.1012028

**Published:** 2026-01-23

**Authors:** Sudhir Kumar, Xueping Fan, Harshita Pattam, Kun Yan, Easton J. Liaw, Jiayi Ji, Emily Zaltz, Paul Song, Yuqiao Jiang, Yuriko Nishizaki, Yujiro Higashi, Chen-Leng Cai, Weining Lu

**Affiliations:** 1 Nephrology Section, Department of Medicine, Boston University Chobanian & Avedisian School of Medicine, Boston Medical Center, Boston, Massachusetts, United States of America; 2 Laboratory of Biochemistry, Department of Health and Pharmaceutical Sciences, Yokohama University of Pharmacy, Yokohama, Kanagawa, Japan; 3 Institute for Developmental Research, Aichi Human Service Center, Kasugai, Aichi, Japan; 4 Center for Developmental and Regenerative Medicine, Guangzhou Institutes of Biomedicine and Health, Chinese Academy of Sciences, Guangzhou, China; University of California San Diego, UNITED STATES OF AMERICA

## Abstract

Mowat-Wilson Syndrome (MWS) is an autosomal dominant genetic disorder caused by heterozygous mutations or deletions in the Zinc finger E-box-binding homeobox 2 (*ZEB2*) gene. Congenital anomalies of the kidney and urinary tract (CAKUT), including hydroureter and hydronephrosis, have been reported in patients with MWS. However, the role of the *ZEB2* gene in urinary tract development and the cellular and molecular mechanisms underlying the CAKUT phenotypes in MWS remain unknown. In this study, we examined ZEB2 expression in the developing mouse ureter and generated *Zeb2* ureteral mesenchyme-specific conditional knockout mice (*Zeb2* cKO) by crossing *Zeb2* floxed mice with *Tbx18*Cre^+^ mice. The urinary tract of *Zeb2* cKO mice and their wild-type littermates was analyzed for morphological and histological changes. Our results show that ZEB2 is expressed in TBX18^+^ ureteral mesenchymal cells during mouse ureter development. Deleting *Zeb2* in these cells caused hydroureter and hydronephrosis, indicating obstructive uropathy. Cellular and molecular marker analysis revealed that the TAGLN^+^ACTA2^+^ ureteral smooth muscle cell (SMC) layer was absent in *Zeb2* cKO mice. In contrast, the tunica adventitia cell layer was significantly expanded compared to controls. At the molecular level, *Zeb2* cKO mice had significantly decreased TBX18 expression but increased SOX9 expression in the developing ureter compared to wild-type controls. Our findings demonstrate that ZEB2 is crucial for normal ureteral SMC differentiation during ureter development. Additionally, our study suggests that MWS patients may have abnormal ureteral SMC development, which contributes to the abnormalities of the urinary tract.

## Author summary

Hydroureter and hydronephrosis are common congenital anomalies with a high incidence (1:100–1:500) that can lead to obstructive uropathy and renal failure in the pediatric population. Mowat-Wilson Syndrome (MWS), caused by heterozygous mutations in the *ZEB2* gene, is a genetic disorder characterized by multiple congenital developmental defects, including hydroureter and hydronephrosis. However, the molecular function of ZEB2 in ureter development and the pathogenesis of hydroureter and hydronephrosis remain unknown. Here, we demonstrate that ZEB2 is expressed in developing ureteral mesenchymal cells, and the deletion of ZEB2 in these cells leads to the loss of ureteral smooth muscle cells, which are replaced by tunica adventitia cells, resulting in a hydroureter and hydronephrosis phenotype. Hence, our work not only demonstrates the critical role of ZEB2 in ureter development but also provides insight into the molecular mechanisms underlying urinary tract anomalies in MWS patients.

## Introduction

The mammalian ureters consist of two tissue compartments, each containing three cell types. The outer mesenchymal compartment has an outer layer of fibrocytes of the tunica adventitia, the middle smooth muscle cell (SMC) layer, and the inner lamina propria layer. The inner compartment is composed of epithelial cell layers [1–4]. The three cell layers of the mesenchymal compartment are derived from the TBX18^+^ ureteric mesenchymal progenitors [4]. Between E15.5 and E16.5, a subset of TBX18^+^ ureteric mesenchymal cells differentiates into the middle SMC layer [4,5]. Any abnormality in ureteral SMC development impairs the formation of a functional ureter, leading to ureter dilation, hydroureter, hydronephrosis, and obstructive uropathy [1–3]. Hydroureter and hydronephrosis have a high incidence (1:100–1:500) in the pediatric population and are a leading cause of obstructive uropathy and renal failure [6,7].

Mowat-Wilson Syndrome (OMIM #235730; MWS), caused by heterozygous mutations or deletions in the Zinc finger E-box-binding homeobox 2 (*ZEB2)* gene, is a multiple congenital anomaly syndrome characterized by an atypical face, moderate-to-severe intellectual retardation, epilepsy, and variable congenital malformations, including Hirschsprung disease (HSCR), congenital heart defects, agenesis of the corpus callosum, and eye anomalies [8–14]. Over 50% of MWS patients have urogenital/renal anomalies, including duplex kidney, pelvic kidney, vesicoureteral reflux (VUR), hydroureter, and hydronephrosis [15–18]. Although many MWS patients develop hydroureter and hydronephrosis, the molecular function of ZEB2 in ureter development and the pathogenesis of CAKUT have not been studied.

ZEB2 is a transcription factor and a member of the zinc-finger E-box–binding (ZEB) protein family. ZEB2 protein plays a crucial role in determining cell fate and the differentiation of embryonic stem cells, including hematopoietic progenitors and neural progenitor cells [19–28]. Previously, we demonstrated that loss of the *Zeb2* gene in nephron progenitors leads to glomerulocystic kidney and renal failure [29]. We also found that the *Zeb2* gene controls the differentiation of FOXD1 + stromal progenitor cells, and the loss of ZEB2 in kidney stromal progenitors leads to kidney fibrosis [30]. In this study, we hypothesize that ZEB2 also plays a crucial role in ureter development, and the loss of the *Zeb2* gene in the developing ureter results in abnormal ureter morphogenesis, hydroureter, and hydronephrosis. To test this hypothesis, we analyzed ZEB2 expression in the developing mouse ureter and generated ureter-specific *Zeb2* conditional knockout mice to investigate its role in ureter development.

Our results show that ZEB2 is highly expressed in the TBX18^+^ ureteral mesenchymal cells. Deletion of the *Zeb2* gene in mouse ureteral mesenchymal cells using *Tbx18Cre*^+^ led to hydroureter and hydronephrosis. Cellular marker analyses showed that ureteral mesenchyme-specific *Zeb2* conditional knockout mice (also called *Zeb2* cKO in this paper) had no TAGLN^+^ACTA2^+^ ureteral smooth muscle cells (SMCs) but instead had an expanded layer of FOXD1^+^POSTN^+^ ureteral tunica adventitia cells during early ureter development. At the molecular level, we found that *Zeb2* knockout ureters had reduced TBX18 expression with an upregulated SOX9 expression, which are critical transcriptional factors for ureteral SMC development. Our results indicate that ZEB2 is essential for the differentiation of ureteral SMCs, and loss of ZEB2 leads to depletion in the ureteral SMC layer that is replaced by the tunica adventitia layer, eventually leading to hydroureter, hydronephrosis, obstructive uropathy, and renal failure.

## Result

### ZEB2 is expressed in TBX18^+^ ureteral mesenchymal cells in the developing ureter

**Fig 1:**
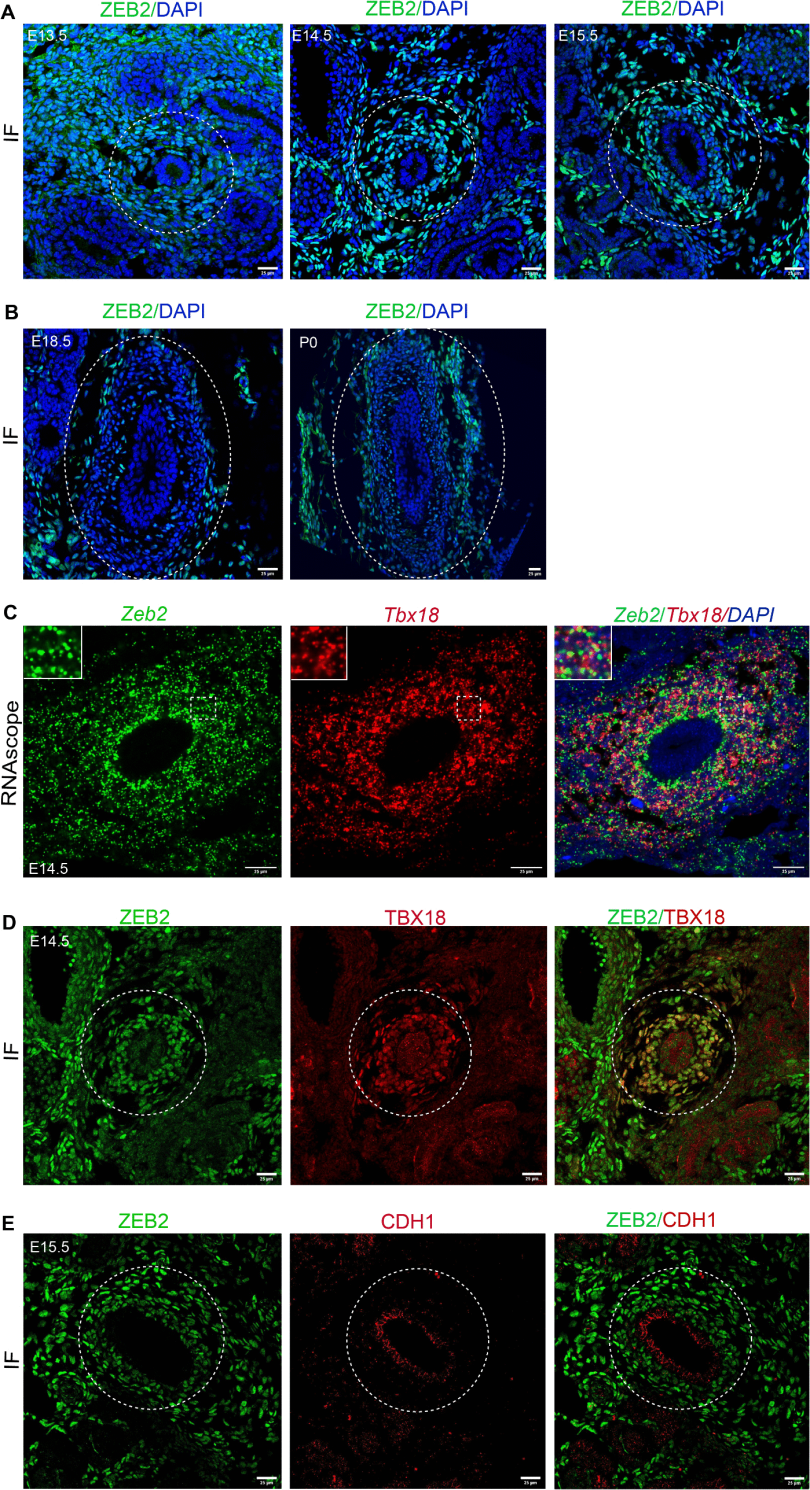
ZEB2 is expressed in TBX18^+^ ureteral mesenchymal cells in the developing ureter. **(A)** ZEB2 (green) expression in the proximal section of the developing ureter at the stages (E13.5, E14.5, E15.5) in the wild-type mouse. **(B)** The panel shows ZEB2 expression at E18.5 and P0. **(C)** Analysis of *Zeb2* expression at E14.5 and co-localization with *Tbx18* by RNAscope in situ hybridization. **(D)** ZEB2 (green) is co-localized with ureteral mesenchymal progenitor cell marker TBX18 (red) in the mouse developing ureter at E14.5. **(E)** ZEB2 (green) is not co-localized with CDH1 (urothelial cell marker) in the developing mouse ureter. Dotted lines encircle the ureter. Dotted box regions in panel C show an enlarged inset in panel **C.** IF: immunofluorescence. Scale bars: 25 µm **(A-E)**. Number of animals analyzed: n = 3 per group.

To elucidate the role of *Zeb2* during ureter development, we first examined the expression of ZEB2 using immunostaining. We found that ZEB2 is highly expressed in the undifferentiated mesenchyme cells in the developing mouse ureters (Fig 1A-1B). We also confirmed *Zeb2* expression in the ureter by in situ hybridization (RNAscope) (Fig 1C). To determine the cell-specific expression of ZEB2, we performed marker analysis by double immunostaining of ZEB2 with ureteral mesenchymal cell marker TBX18. Immunostaining and RNAscope analysis showed that ZEB2 is colocalized with TBX18 (Fig 1C-1D). We also examined ZEB2 expression in urothelial cells by performing double immunostaining of ZEB2 and E-cadherin-1 (CDH1, urothelial cell marker) and found that ZEB2 was not expressed in these cells (Fig 1E). These results suggest that ZEB2 is expressed mainly in TBX18^+^ ureteral mesenchymal cells during ureter development.

### Loss of *Zeb2* in TBX18^+^ ureteral mesenchymal cells leads to hydroureter, hydronephrosis, and obstructive uropathy

**Fig 2:**
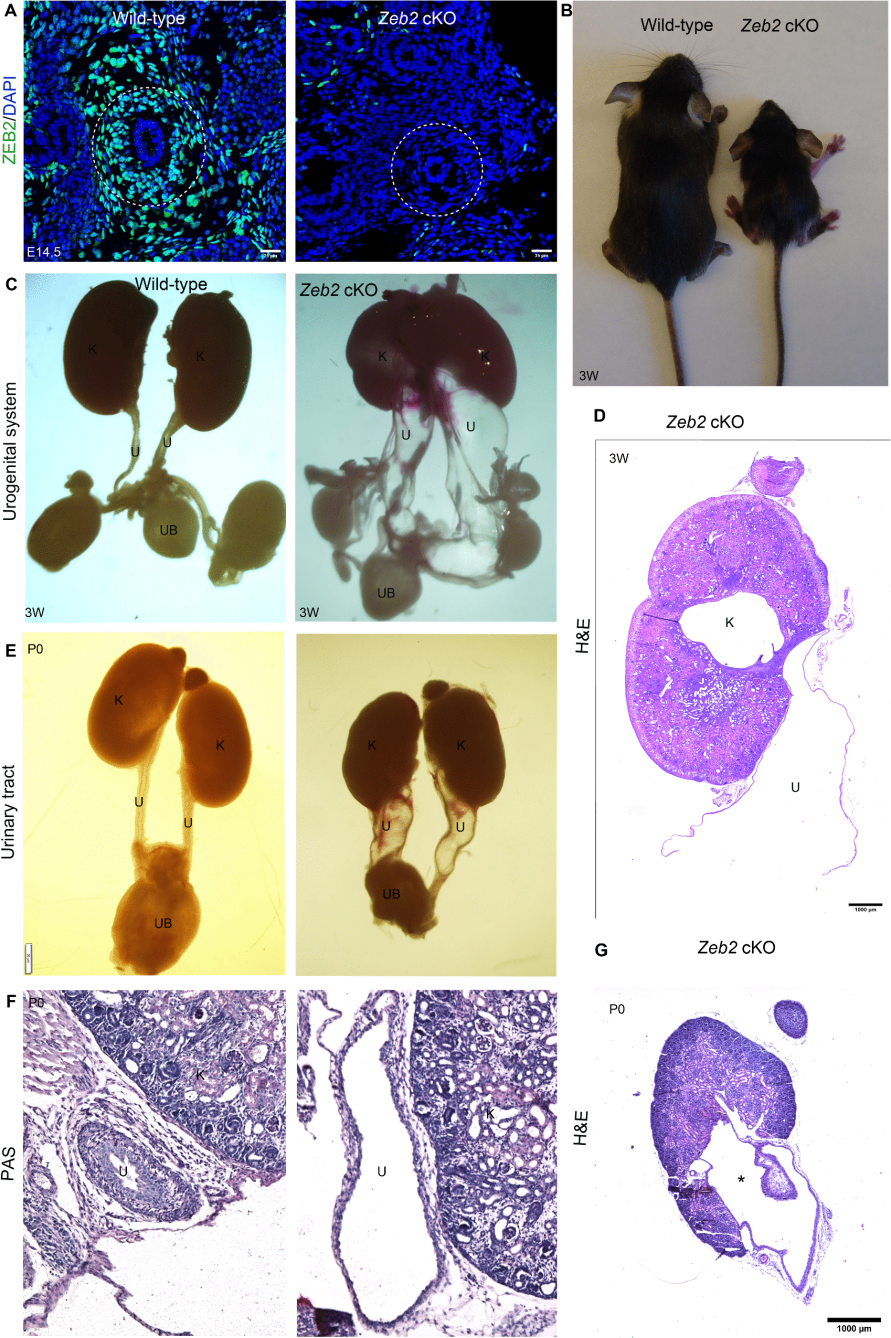
Loss of *Zeb2* in TBX18^+^ ureteral mesenchymal cells leads to hydroureter, hydronephrosis, and obstructive uropathy. **(A)** ZEB2 (green) expression is absent in *Zeb2*^flox/flox^;*Tbx18Cre*^+^ knockout mice (*Zeb2* cKO) as compared to wild-type controls. **(B)** Three-week-old *Zeb2* cKO mice were runted with smaller body sizes compared to wild-type control littermates. (**C**) 3-week-old *Zeb2* cKO mice developed hydroureter and hydronephrosis. **(D)** H&E staining of the kidney of a 3-week-old *Zeb2* cKO mouse showing a dysplastic kidney with a dilated ureter. **(E)** Newborn (P0) *Zeb2* cKO mice develop hydroureter and hydronephrosis. **(F)** Histology of a newborn (P0) *Zeb2* cKO mouse ureter with hydroureter as compared to wild-type littermate controls. **(G)** Histology of a newborn (P0) *Zeb2* cKO mouse kidney with hydronephrosis (*). u: ureter, k: kidney, UB: urinary bladder. Dotted lines encircle the ureter. Scale bars: 25 µm **(A)**; 1000 µm **(D and G)**. Number of animals analyzed: n = 5 per group.

To understand the function of ZEB2 in TBX18^+^ ureteral mesenchymal cells, we generated *Zeb2* conditional knockout mice, *Zeb2*^flox/flox^;*Tbx18Cre*^+^ (*Zeb2* cKO), by crossing *Zeb2* floxed mice [31] with *Tbx18Cre*^*+*^ mice [32]. The deletion of *Zeb2* in ureteral mesenchymal cells was confirmed by immunostaining, which revealed the absence of ZEB2 signals in the ureter of *Zeb2* cKO mice compared to wild-type controls (Fig 2A). *Zeb2* cKO mice were born in the Mendelian ratio (Table 1), but most homozygous *Zeb2* cKO mice died within a few hours after birth, and only a few survived up to 3 weeks of age (Table 1). The 3-week-old *Zeb2* cKO mice were runted and hunched (Fig 2B) and developed hydroureter and hydronephrosis phenotypes (Fig 2C-2D). Some *Zeb2* cKO mice were found to have high-grade hydroureter, which may be classified as megaureters. We also analyzed newborn mice (P0) and found that homozygous *Zeb2* cKO newborn pups also developed hydroureter and hydronephrosis phenotypes as well (Fig 2E-2G). The high-grade hydroureter in *Zeb2* cKO mice prompted us to investigate whether the ureterovesical junction (UVJ) is similarly affected. We examined the UVJ in newborn *Zeb2* cKO mice and wild-type controls. In morphological and histological analyses, we found that *Zeb2* cKO mice exhibited partial UVJ obstruction compared to wild-type mice (S1A-D Fig). We also analyzed whether hydronephrosis leads to tubule dilations in the kidneys; we found that 3-week-old *Zeb2* cKO mice had significantly dilated tubules (S2 Fig). Taken together, our data suggest that in the absence of ZEB2, ureters do not develop properly, leading to hydroureter and hydronephrosis.

**Table 1:** *Zeb2* cKO mice were born in a Mendelian ratio but died after birth.

Genotype	3 weeks (*n* = 79)		P0 (*n* = 78)	
	Observed	Expected	Observed	Expected
*Zeb2*^flox/flox^*;Tbx18Cre*^+^ (*Zeb2* cKO)	4 (5%)	25%	20 (26%)	25%
*Zeb2*^flox/ *+*^ *;Tbx18Cre*^+^ (*Zeb2* het)	28 (35%)	25%	22 (28%)	25%
*Zeb2*^flox/flox^*;Tbx18Cre*^-^ (WT)	23 (29%)	25%	18 (23%)	25%
*Zeb2*^Flox*/ +*^ *;Tbx18Cre*^-^ (WT)	24 (31%)	25%	18 (23%)	25%

### Ureteral mesenchymal progenitors fail to differentiate into smooth muscle cells in the absence of ZEB2

**Fig 3:**
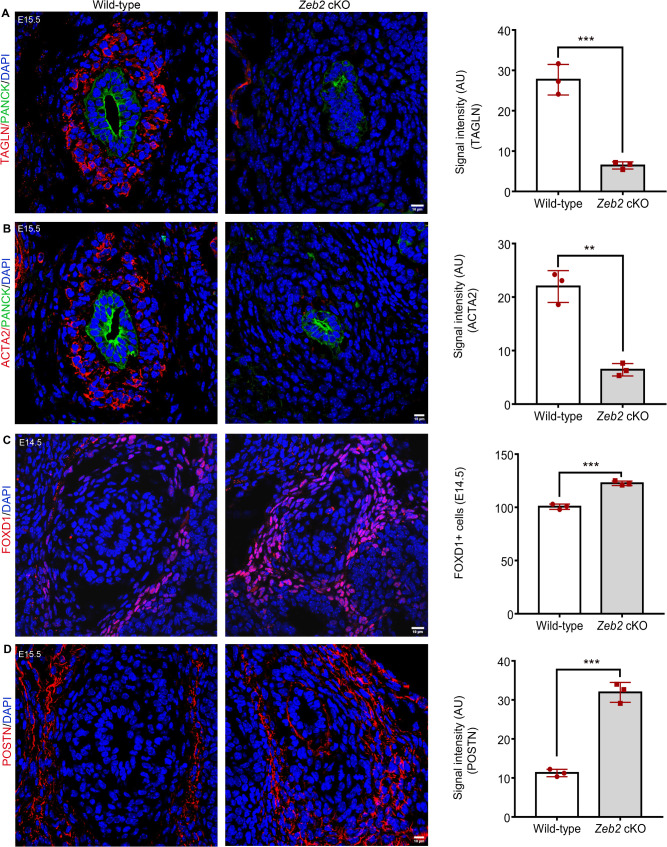
Ureteral mesenchymal progenitors fail to differentiate into smooth muscle cells in the absence of ZEB2. **(A, B)** Ureteral smooth muscle cells are formed in the wild-type mice at E15.5, specified by smooth muscle markers TAGLN (**A**) and ACTA2 **(B)**. There are no TAGLN^+^ and ACTA2^+^ ureteral smooth muscle cells in *Zeb2* cKO mice. Pan-cytokeratin (Pan-cyto) is used to mark urothelial cells. **(C, D)** Compared to wild-type littermate controls, ureteral tunica adventitia cell layers labeled by POSTN^+^ and FOXD1^+^ markers are expanded in *Zeb2* cKO mice. Scale bars: 10 µm **(A-D)**. Number of animals analyzed: n = 3 per group. Sections of the proximal ureter region were analyzed. Values are expressed as mean ± SEM. Student’s *t*-test is used for statistical significance. **P < 0.01, ***P < 0.001.

**Fig 4:**
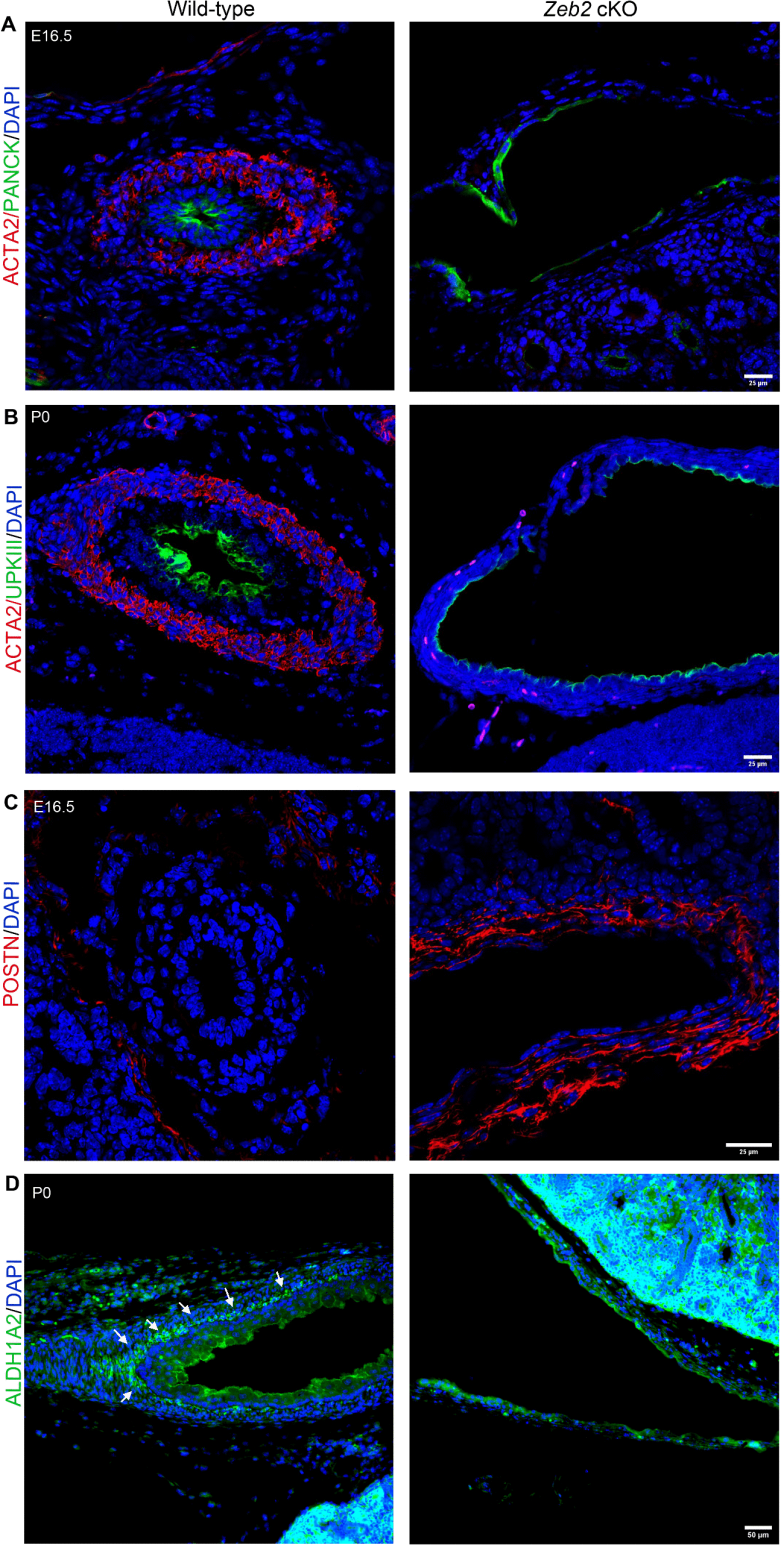
*Zeb2* cKO mice did not show delayed SMC development. **(A-B)** Immunofluorescent staining of ACTA2 (smooth muscle cell marker) at the proximal ureter sections at E16.5 and P0 in the *Zeb2* cKO mice and wild-type controls. Pan-cytokeratin (Pan-cyto) and uroplakin III (UPKIII) were used to mark urothelial cells. No SMC layer was found in *Zeb2* cKO mice as compared to the wild-type controls. **(C)** Immunofluorescent staining of POSTN (tunica adventitia cell marker) at the proximal ureter sections at E16.5 in the *Zeb2* cKO mice and wild-type controls. **(D)** Immunofluorescent staining of ALDH1A2 (lamina propria cell marker, marked by arrows) at the proximal ureter sections at P0 in the *Zeb2* cKO mice and wild-type controls. Scale bars: 25 µm **(A-C)**, 50 µm **(D)**. Number of animals analyzed: n = 4 per group.

During ureter development, a subset of TBX18 + ureteral mesenchymal cells differentiate into smooth muscle cells (SMCs), which are crucial for functional ureter formation and prevent ureter dilation after urine production [2,5]. To check the SMC formation in the developing ureter of *Zeb2* cKO mice, we examined two established SMC markers, the actin alpha 2, smooth muscle (ACTA2, Gene ID: 59), and transgelin (TAGLN, Gene ID: 6876) [4,33]. Pan-cytokeratin was used to demarcate the urothelial cells [34]. Analysis at the proximal ureter level revealed that wild-type mice formed a normal ACTA2^+^TAGLN^+^ SMC layer at E15.5; however, this layer was absent in age-matched *Zeb2* cKO mice (Fig 3A-3B, S1 Data). TBX18^+^ ureteral mesenchymal cells also give rise to the tunica adventitia layer that expresses marker genes forkhead box D1 (FOXD1, Gene ID: 2297) and periostin (POSTN, Gene ID: 10631) [4]. We, therefore, asked if there is any change in the development of the tunica adventitia ureter layer in the absence of ZEB2. We examined the tunica adventitia cells by specific antibody immunostaining and found an expanded FOXD1^+^POSTN^+^ tunica adventitia layer in the developing ureter of *Zeb2* cKO mice as compared to their wild-type littermate controls (Fig 3C-3D, S1 Data). To determine if there is delayed SMC differentiation in *Zeb2* cKO mice, we analyzed the SMC layer development at later stages (E16.5 and P0). We found that the SMC layer did not develop in *Zeb2* cKO mice at these stages either (Fig 4A-4B). However, the adventitia cell layer was found to be expanded in *Zeb2* cKO mice, as evidenced by POSTN immunostaining at E16.5 (Fig 4C). We also analyzed two additional markers for the tunica adventitia, collagen type 1 alpha 2 (COL1A2) and BMP-binding endothelial regulator (BMPER) [33]. In the wild-type mice, expression of BMPER was restricted to tunica adventitia and urothelial cells; however, there was an expansion of BMPER expression in the *Zeb2* cKO mice, and a significant upregulation was observed in the mesenchymal cells (S3A Fig.). This data suggests that ZEB2 may regulate BMP signaling during ureter development [35], as has been reported in neural crest formation. Adventitia marker COL1A2 was also found to be upregulated in *Zeb2* cKO mice compared to wild-type controls (S3B Fig), We also analyzed the lamina propria development in *Zeb2 cKO* mice with ALDH1A2 immunostaining [36]. We found that there is absence of ALDH1A2 expression in the ureters of newborn *Zeb2* CKO mice as compared to controls (Fig 4D), suggesting that the lamina propria did not differentiate properly in these mice.

**Fig 5:**
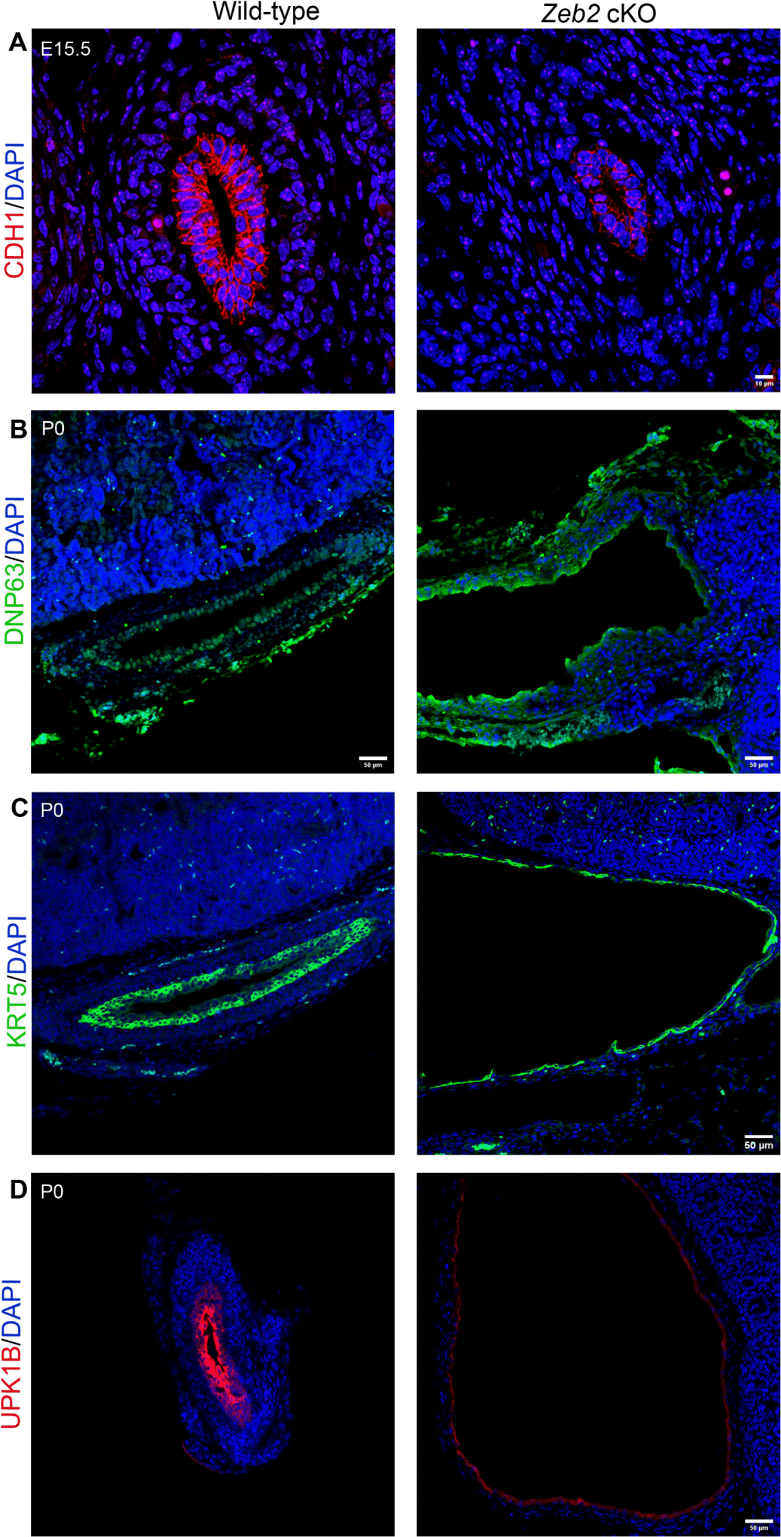
Analysis of urothelium cell differentiation on proximal ureters in *Zeb2* cKO mice. **(A)** CDH1^+^ urothelium cells are underdeveloped in *Zeb2* cKO mice compared to wild-type littermate controls. **(B-D)** Immunofluorescent staining of ∆NP63 (basal and intermediate cell marker), KRT5 (basal cell marker), and UPK1B (intermediate and superficial cell marker), at the proximal ureter sections in P0 *Zeb2* cKO mice and wild-type controls. Urothelial cell differentiation was found to be unaffected in the ureters of *Zeb2* cKO mice. Scale bars: 10 µm **(A)**, 50 µm **(B-D)**. Number of animals analyzed: n = 4 per group.

To check if urothelial cell differentiation was also disturbed in *Zeb2* cKO developing ureter, we analyzed the expression of E-cadherin (CDH1, urothelial cell stratification marker), KRT5 (basal cell marker), UPK1B (intermediate and superficial cell marker), and ∆NP63 (basal and intermediate cell marker) [4]. We found that *Zeb2* cKO mice had significantly reduced expression of CDH1 and ∆NP63 in the urothelial cells (Fig 5A-5B). However, other urothelial cell markers (KRT5 and UPK1B) were found to be unaffected in the ureters of *Zeb2* cKO mice, suggesting KRT5^+^ and UPK1B^+^ urothelial cells were present in these mice (Fig 5C-5D). These data indicate that urothelial cell stratification was affected in *Zeb2* cKO mice. The defective stratification of the urothelial cells may be due to abnormal ureteral mesenchymal differentiation, as the ureteral mesenchymal cells crosstalk with the urothelial cells during ureter development [37]. Taken together, our data suggest that ZEB2 is essential for the normal differentiation of the ureteral mesenchymal cells into SMCs to form functional ureters during development, and loss of ZEB2 causes the absence of SMCs, which leads to hydroureter and hydronephrosis.

### Loss of ZEB2 alters the expression of key transcription factors required for proper ureteral SMC differentiation and development

**Fig 6:**
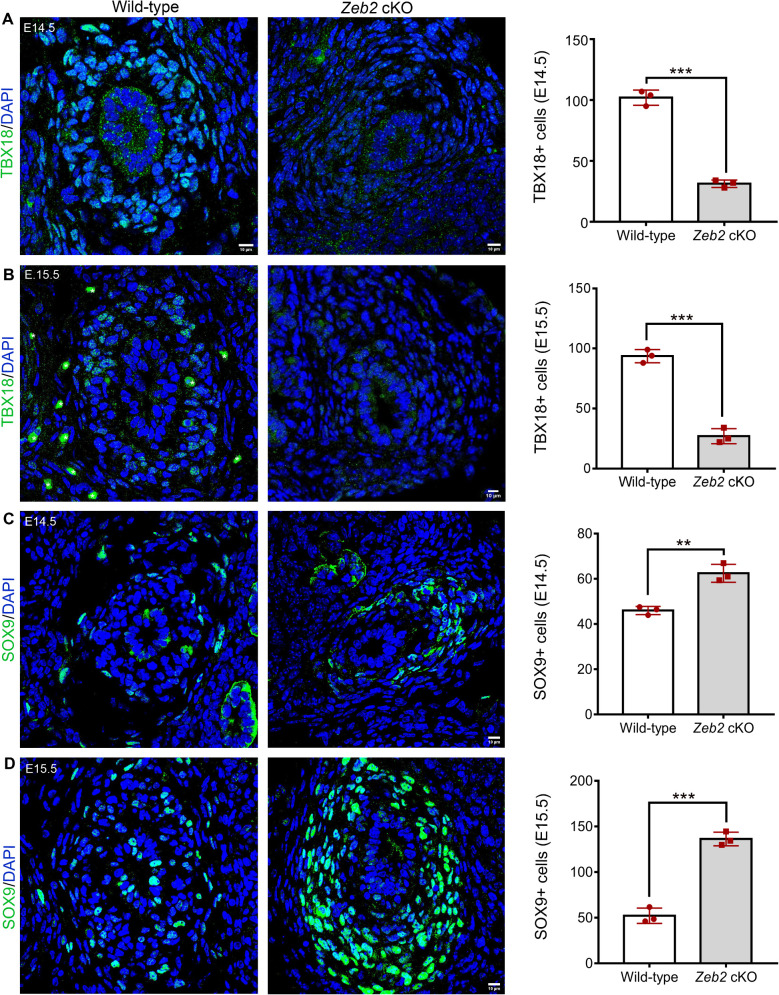
Loss of ZEB2 altered the expression of key factors required for proper ureteral SMC differentiation and development. **(A-B)** Immunostaining of TBX18 on the proximal ureter sections shows decreased TBX18 (green) expression in ureteral mesenchymal progenitors at E14.5 and E15.5 in *Zeb2* cKO mice compared to wild-type controls. We excluded artifact staining in panel B in wildtype sections (marked by *) in the analysis. **(C-D)** Immunostaining of SOX9 on the proximal ureter sections shows increased SOX9 (green) expression in ureteral mesenchymal progenitors at E14.5 and E15.5 in *Zeb2* cKO mice compared to wild-type controls. Scale bars: 10 µm **(A-D)**. Number of animals analyzed: n = 3 per group. Values are expressed as mean ± SEM. Student’s *t*-test is used for statistical significance. **P < 0.01, ***P < 0.001.

**Fig 7:**
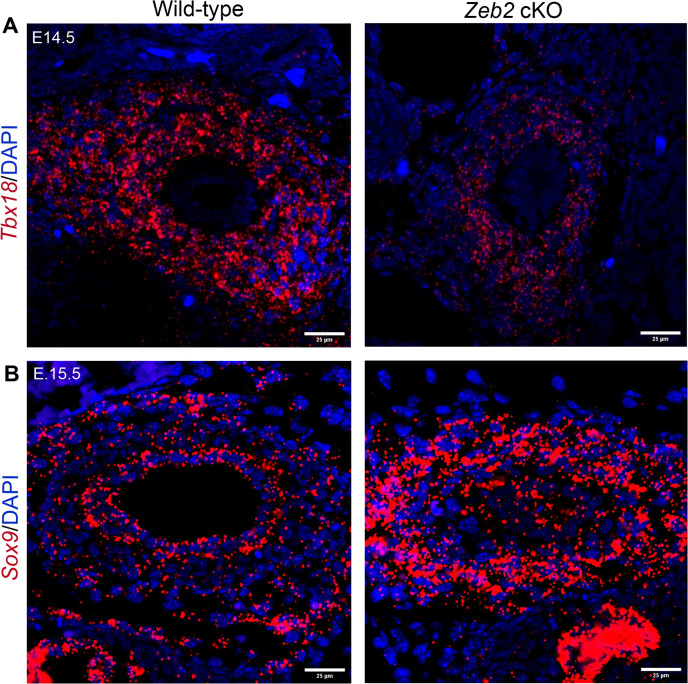
RNAscope (in situ hybridization) analysis on proximal ureters for *Tbx18* and *Sox9* expression in *Zeb2* cKO mice. **(A)** RNAscope of *Tbx18* mRNA on the proximal ureter sections shows decreased *Tbx18* (red) expression in ureteral mesenchymal progenitors at E14.5 in *Zeb2* cKO mice compared to wild-type controls. **(B)** RNAscope of *Sox9* mRNA on the proximal ureter sections shows increased *Sox9* (red) expression in ureteral mesenchymal progenitors at E15.5 in *Zeb2* cKO mice compared to wild-type controls. Scale bars: 25 µm **(A-B)**. Number of animals analyzed: n = 3 per group.

Transcription factor *Tbx18* is the earliest marker for the ureteric mesenchyme, which specifies it and gives rise to all the mesenchymal cells of the ureters [5,38]. Previously, it has been shown that TBX18 (Gene ID: 9096) and SOX9 (Gene ID: 6662) are essential for ureteral SMC layer development, and loss of TBX18 or SOX9 leads to the absence of ureteral SMCs, resulting in hydroureter [5,39]. We performed TBX18 and SOX9 immunostaining on the proximal ureter sections and examined their expressions to understand the molecular and phenotypic changes in the *Zeb2* cKO ureters. We found a significant reduction in TBX18 expression in the *Zeb2* cKO mouse ureter at E14.5 and E15.5 (Fig 6A-6B, S2 Data). Interestingly, SOX9 expression was significantly upregulated in the E15.5 *Zeb2* cKO mouse ureter as compared to their wild-type littermate controls (Fig 6C-D, S2 Data). However, SOX9 was significantly lower in epithelial cells at E15.5 in *Zeb2* cKO mice as compared to controls, suggesting that epithelial cells did not differentiate properly as we mentioned before. We also examined the expression of these markers using RNAscope (in situ hybridization) and confirmed a reduction in *Tbx18* expression, and an increase in *Sox9* expression in the *Zeb2* cKO mouse ureter compared to the wild-type control (Fig 7A-7B). These data suggest that loss of ZEB2 perturbs the expression of key transcription factors such as TBX18 and SOX9, which are critical for proper ureteral SMC differentiation and ureter development.

As *Zeb2* cKO mice had an expanded FOXD1^+^POSTN^+^ tunica adventitia layer and increased SOX9 expression. We examined whether markers of tunica adventitia (FOXD1 and POSTN) colocalize with SOX9. We performed RNAscope and immunostaining at the proximal ureter level at different embryonic stages and found that *Foxd1* and *Sox9* colocalize at the same level on the outer side in *Zeb2* cKO mice compared to wild-type controls. However, *Zeb2* cKO mice had expanded POSTN expression that colocalized with SOX9 as compared to wild-type controls (S4A-C Fig). To determine if these expression alterations affect the proliferation of ureteral cells, we examined the cell proliferation by performing phosphohistone H3 (PHH3) immunostaining on the proximal ureter sections at E14.5 and E15.5. We did not observe any changes in the cell proliferation in *Zeb2* cKO mice as compared to wild-type control (S5A-B Fig, S3 Data). We also investigated whether mesenchymal cell condensation was disturbed in *Zeb2* cKO mice, as TBX18 is required for ureteral mesenchymal cell condensation [5]. Histological examination showed that ureteral mesenchymal cells were abnormally condensed, compacted, and disorganized in *Zeb2* cKO pups as compared to the wild-type controls (S6 Fig). These data suggest that loss of ZEB2 leads to mesenchymal patterning defects, which cause SMC loss in *Zeb2* cKO mice ureters.

## Discussion

**Fig 8:**
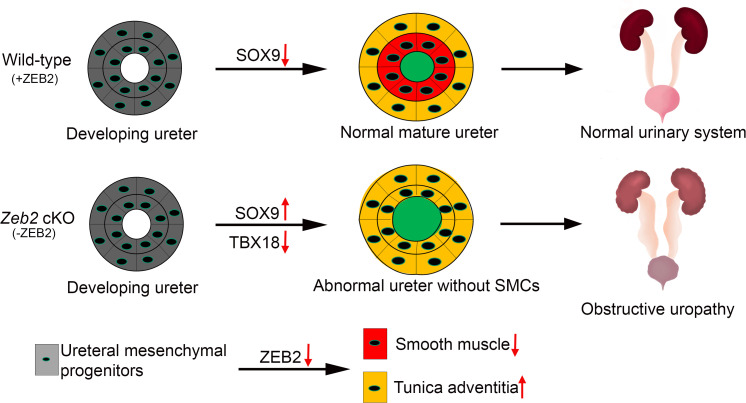
Proposed Model: Transcription factor ZEB2 is essential for ureteral smooth muscle cell differentiation during ureter development.

Mowat-Wilson Syndrome (OMIM #235730; MWS) is caused by heterozygous mutations or deletions in the *ZEB2* gene, and more than half of MWS patients have urinary tract anomalies, including duplex kidney, pelvic kidney, VUR, and hydronephrosis [8–18,27]. In this report, we found that ZEB2 is expressed in the TBX18^+^ ureteral mesenchymal cells, and deletion of *Zeb2* by *Tbx18Cre*^+^ leads to hydroureter, hydronephrosis, and obstructive uropathy. We found that *Zeb2* cKO mice do not develop the ureteral SMC layer but have an expanded layer of tunica adventitia cells*.* At the molecular level, we found that loss of ZEB2 leads to a reduced expression of transcription factor TBX18 but an increased expression of SOX9 in the ureteral mesenchymal cells, both of which are critical for ureteral SMC formation (Fig 8). These findings, hence, provide new molecular insights into the novel role of ZEB2 in the differentiation and development of ureteral SMCs.

In recent years, progress has been made in understanding the molecular signals regulating ureteral SMC development [2,3,40]. The factors controlling ureter development have been reported in a recent review and some are major factor are Sonic Hedgehog signaling, WNT signaling, *Tbx18*, *Sox9*, *Bmp4*, *Six1, Foxf1, Smo*, and *Smad4* [1–3,5,33,37,40–46]. *Tbx18* regulates the specification and development of the ureteric mesenchyme, and *Tbx18*^*-/-*^ knockout mice do not develop the SMC layer [5]. SOX9 is also crucial for ureteral mesenchymal cell differentiation, and loss of SOX9 also leads to the absence of SMCs [39]. Interestingly, *Zeb2* cKO mice also failed to develop SMCs and exhibited decreased expression of TBX18, but increased expression of SOX9 in the developing ureteral mesenchymal cells. Previously, it has been demonstrated that ZEB2 antagonizes the inhibitory effect of SOX2, a member of the family of SOX proteins, on Schwann cell differentiation and myelination [47,48]. ZEB2 also controls SMC phenotypic transition through chromatin remodeling, and loss of ZEB2 leads to differentially open chromatin for mature SMC regulatory markers such as *Cnn1*, *Myh11*, and *Myocd*, and chondrogenesis-related genes such as *Spp1*, *Sox9*, and *Col2a1* [49]. Our findings in this study discover ZEB2 as a novel ureter regulatory gene and suggest that ZEB2 might antagonize the inhibitory effect of SOX9 during ureteral SMC differentiation and development by altering chromatin accessibility. Further studies are needed to elucidate how ZEB2 interacts with TBX18 and SOX9 during ureter development as well as the downstream genes that ZEB2 regulates in the developing ureter.

We also observed an expansion of the ureteral tunica adventitia cells in the *Zeb2* cKO mice in addition to the loss of SMCs in the developing ureter. TBX18^+^ mesenchymal progenitors can subdivide into the inner and outer mesenchymal cells during ureter development. The inner mesenchymal cells further differentiate into the functional SMC layer to support ureter unidirectional peristalsis and prevent ureter dilation; in contrast, the outer compartment of TBX18^+^ mesenchymal cells gives rise to tunica adventitia cells [4]. Expansion of tunica adventitia cells in *Zeb2* cKO mice might be due to failure of the TBX18^+^ mesenchymal progenitors to separate into inner and outer compartments in the absence of ZEB2. As a result, all TBX18^+^ mesenchymal progenitors differentiate into tunica adventitia cells. Alternatively, loss of the SMC layer in the *Zeb2* cKO may stimulate the expansion of the ureteral tunica adventitia layer. Thirdly, this unexpected cellular phenotype of expanded ureteral tunica adventitia layer could also be driven by upregulated SOX9 expression in the ureteral mesenchymal cells, as prolonged expression of SOX9 in the ureteral mesenchyme reduces smooth muscle gene expression and increases the expression of extracellular matrix (ECM) components, as previously reported [39]. Lastly, a recent study also suggests that SMC-specific loss of ZEB2 results in an inability to turn off contractile programming, leading to SMCs transitioning into a fibroblast-like phenotype [49]. Further studies are needed to confirm if ZEB2 specifically regulates early ureteral mesenchymal cell specification during ureter development and if the expanded ureteral tunica adventitia layer phenotype is merely a compensatory response to SMC loss in *Zeb2* cKO mice.

In past years, much progress has been made in understanding the role of ZEB2 during nervous system development [22,50]. However, the role of ZEB2 in the urinary system is poorly understood, despite more than 50% of MWS patients being reported to have urinary tract anomalies. Our current work not only demonstrates the critical role of ZEB2 in ureter development but also provides molecular genetic insights into some aspects of MWS-associated phenotypes in the urinary tract.

In summary, we found that loss of ZEB2 in ureteral mesenchymal cells leads to ureteral SMC loss, which eventually causes hydroureter, hydronephrosis, and obstructive uropathy (Fig 8). Our data suggest that ZEB2 is essential for ureteral SMC differentiation and development during ureter development. Our study also indicates that urinary tract anomalies are the primary phenotype in MWS patients and that MWS patients should be evaluated for urinary tract disorders such as hydroureter, hydronephrosis, and obstructive uropathy.

## Materials and methods

### Ethics statement

All animal experiments were performed according to the guidelines issued by the Institutional Animal Care and Use Committee of Boston University Medical Campus at Boston University (approved IACUC protocol number: PROTO201800056_TR01).

### Animals

*Zeb2* floxed mice [31] and *Tbx18Cre*^*+*^ mice [32] were genotyped using primers previously reported. The ZEB2-EGFP knock-in reporter mouse was studied previously [51]. Eight-week-old *Zeb2*^flox/+^;*Tbx18Cre*^*+*^ mice were bred with *Zeb2*^flox/flox^ mice to generate *Zeb2*^flox/flox^;*Tbx18Cre*^*+*^ homozygous conditional knockout mice. All mice had free access to drinking water and a standard rodent diet ad libitum. Animals of both sexes with mixed C57BL/6 x 129 genetic backgrounds were used in this study. Wild-type littermates were used as controls. Timed-pregnant females were confirmed by a semen plug and designated at embryonic day 0.5 (E0.5) of gestation and were separated from the breeding cage.

### Tissue preparation and histology

Gross urinary tracts of newborn *Zeb2* cKO mice and wild-type littermates were dissected and imaged with an Olympus inverted dissecting microscope (Olympus, USA). For histological analysis, urinary tracts were fixed in 4% paraformaldehyde or 10% neutral buffered formalin at 4°C overnight and then processed for paraffin embedding using standard protocols. Serial kidney sections were cut and stained using a Periodic acid Schiff (PAS) stain kit (# 24200, Polysciences, Inc., USA) and Hematoxylin and Eosin (H&E) Stain Kit (H-3502, Vector Labs, USA), according to the manufacturer’s instructions. For immunohistochemistry, paraffin-embedded tissue sections were deparaffinized and steamed for 30 minutes in an antigen retrieval solution (IHC-Tek antigen retrieval solution, IW-1100, IHCworld LLC, USA), or antigen retrieval buffer (100X EDTA Buffer, ab93680, pH 8.0, abcam, USA). Slides were examined with an Olympus upright light microscope and photographed using an Olympus DP72 digital camera.

### Immunofluorescence staining and confocal microscopy

Embryos were harvested from timed-pregnant females at embryonic day (E) 13.5, 14.5, 15.5, 16.5, E18.5, and P0. Whole embryos were fixed in 4% formaldehyde overnight at 4°C followed by incubation in 30% sucrose overnight at 4°C, embedded in optimal cutting temperature (OCT) compound (Tissue-Tek, Sakura Finetek), and cryosectioned at 8–10 μm. Frozen sections were permeabilized with PBS containing 0.1% Triton X-100 for 10 minutes and blocked in 5% serum-blocking buffer or background buster (NB306, Innovax biosciences, USA) for 1 hour at room temperature. Primary antibodies were incubated overnight at 4°C, followed by secondary antibodies at room temperature for 1 hour. Following primary antibodies were used: ZEB2 (1:100, HPA003456; Sigma-Aldrich, USA or SC-271984, Santa Cruz Biotechnology, USA), TBX18 (1:100, ab115262, Abcam, USA), E-cadherin (1:50, Clone 36/610181; BD Biosciences, USA), ACTA2 (1:200, A2547; Sigma-Aldrich, USA), Uroplakin III (1:20, 610108, Progen, Germany), Cytokeratin (1:100, C2562, Sigma-Aldrich, USA), TAGLN (1:200, ab14106, Abcam, USA), ALDH1A2 (1:200, ab75674, Abcam, USA), Cytokeratin 5 (1:100, ab52635, Abcam, USA), UPK1B (1:100, WH0007348M2; Sigma-Aldrich, USA) ∆NP63 (1:100, ab735, Abcam, USA), BMPER (1:100, AF2299, R&D systems, USA), COL1A2 (1:100, 14695–1-AP, Proteintech, USA), PHH3 (1:100, 9701, Cell Signaling Technology, USA), FOXD1 (1:100 dilution, sc-47585; Santa Cruz Biotechnology USA), POSTN (1:100, ab14041, Abcam, USA), SOX9 (1:200, AB5535, Millipore, USA), GFP (GFP-1020, 1:100, Aves Labs, USA or A11122, 1:250; Invitrogen, Carlsbad, CA). Secondary antibodies were goat anti-mouse IgG2a AF594, goat anti-mouse IgG1 AF488, goat anti-rabbit IgG AF488, goat anti-rabbit IgG AF494, and donkey anti-rabbit IgG AF594. Sections were stained with DAPI (4´,6´-´diamidino-2-phenylindole) and mounted in Vectashield antifade mounting medium (H-1000; Vector Labs, USA). Images were captured by a Zeiss LSM 700 confocal microscope (Zeiss, Germany) and analyzed with ImageJ software (National Institutes of Health, USA).

### In situ hybridization (RNAscope) assay

In situ hybridization (RNAscope) assay was performed according to the manufacturer’s instructions using the RNAscope multiplex fluorescent detection kit V2 (Cat No. 323110, Advanced Cell Diagnostics USA) as previously described [30]. Briefly, ureters fixed in 4% paraformaldehyde (PFA) were re-fixed in 4% PFA in 1 × PBS at 4°C for 15 min and rinsed twice in 1 × PBS. The slides were then dehydrated in 50%, 70%, and 100% ethanol for 5 min/each. After that, the ureter tissues were incubated in hydrogen peroxide for 10 min at room temperature, rinsed in water, and then incubated in RNAscope protease IV for 30 min at room temperature, followed by two washes in 1 × PBS. The tissues were then incubated with probes of *Zeb2* (RNAscope Probe- Mm-Zeb2, Cat No. 436391; Advanced Cell Diagnostics USA), *Tbx18* (RNAscope Probe- Mm-Tbx18-C2, Cat No. 515221-C2; Advanced Cell Diagnostics USA), *Sox9* (RNAscope Probe- Mm-Sox9-C2, Cat No. 401051-C2; Advanced Cell Diagnostics USA), and *Foxd1* (RNAscope Probe- Mm-Foxd1-C3, Cat No. 495501-C3; Advanced Cell Diagnostics USA) for 2 hours at 40°C, followed by two washes in 1 × PBS. The probe signal was amplified by incubating with RNAscope multiplex FL V2 Amp 1 for 30 min at 40°C, then twice rinsed in 1 × PBS. Finally, the signal was developed by incubating with RNAscope multiplex FL V2 C1, and subsequently with Opal 570 (FP1488001KT, 1:1000 dilution; Akoya Biosciences, USA). After rinsing twice in 1 × PBS, the slides were counterstained with DAPI and mounted in Prolong Diamond antifade mounting medium (Cat No. P36970; Invitrogen USA). Images were captured using a Zeiss 700 confocal microscope (Zeiss, Germany) and analyzed with the NIH ImageJ software.

### Image quantification

Image quantification was performed in ImageJ on random images (*n* = 3 per group). Cell counter or particle analysis plug-in tools were used to quantify the cell numbers. For intensity quantification, the percentage of total pixel area was measured.

### Statistics

All statistical analyses were performed using GraphPad Prism statistical software (version 7.0, GraphPad, San Diego, CA) with *P* < 0.05 considered statistically significant. Wild-type and *Zeb2* cKO mice group data were compared using a two-tailed unpaired Student’s *t-test*. Data are represented as mean ± SEM. A minimum of three mice were used for each analysis group unless stated otherwise.

### Disclosures

All authors have nothing to disclose.

## Supporting information

S1 Fig Morphological and histological analysis of ureterovesical junction (UVJ) in *Zeb2* cKO mice. (A) Whole urinary tract system of P0 *Zeb2* cKO mice and wild-type controls. (B) Highlighted area in panel A. (C) H&E staining of UVJ in P0 *Zeb2* cKO mice and wild-type controls. *Zeb2* cKO mice. (D) Highlighted area in panel C. *Zeb2* cKO mice had partial obstruction of the UVJ as compared with wild-type control mice. Number of animals analyzed: n = 3 per group. U = ureter, UB = Urinary bladder.
(JPG)

S2 Fig Analysis of kidney tubule dilation in *Zeb2* cKO mice: H&E staining of kidneys of 3-week-old *Zeb2* cKO mice and wild-type controls. *Zeb2* cKO mice had significantly dilated tubules due to hydronephrosis. Number of animals analyzed: n = 3 per group.
(JPG)

S3 Fig Analysis of additional tunica adventitia cell markers. (A) Immunofluorescent staining of BMP-binding endothelial regulator (BMPER, green) and collagen 1a2 (COL1A2, red) at the proximal ureter sections at E15.5 in the *Zeb2* cKO mice and wild-type controls. (B) Immunofluorescent staining of BMP-binding endothelial regulator (BMPER, green) and collagen 1a2 (COL1A2, red) at the proximal ureter sections at E16.5 in the *Zeb2* cKO mice and wild-type controls. Scale bars: 25 µm (A-B). Number of animals analyzed: n = 3 per group.
(JPG)

S4 Fig Co-localization of SOX9 and tunica adventitia cell markers. (A) RNAscope of *Foxd1* (green) and *Sox9* (red) at the proximal ureter sections at E14.5 in the *Zeb2* cKO mice and wild-type controls. (B) RNAscope of *Foxd1* (green) and *Sox9* (red) at the proximal ureter sections at E15.5 in the *Zeb2* cKO mice and wild-type controls. (C) Immunofluorescent staining of SOX9 (green) and POSTN (red) at the proximal ureter sections at E15.5 in the *Zeb2* cKO mice and wild-type controls. The expanded SOX9 expression was marked with arrows in panel C. Scale bars: 25 µm (A-C). Number of animals analyzed: n = 3 per group.
(JPG)

S5 Fig Proliferation analysis in *Zeb2* cKO mice. (A) Immunofluorescent staining of PHH3 at the proximal ureter sections at E14.5 in the *Zeb2* cKO mice and wild-type controls. (B) Immunofluorescent staining of PHH3 at the proximal ureter sections at E15.5 in the *Zeb2* cKO mice and wild-type controls. Scale bars: 25 µm (A-B). Number of animals analyzed: n = 3 per group. Values are expressed as mean ± SEM. Student’s *t*-test is used for statistical significance. N.S. = not significant.
(JPG)

S6 Fig Ureteral mesenchymal cells were not condensed adequately in the absence of ZEB2. (A) H&E staining showing normal condensation of ureteral mesenchymal cells in the wild-type mice at E14.5, but it was compacted and disorganized in *Zeb2* cKO mice. Scale bars: 50 µm. Number of animals analyzed: n = 3 per group.
(JPG)

S1 Data Minimal Data set for Figure 3: Ureteral mesenchymal progenitors fail to differentiate into smooth muscle cells in the absence of ZEB2. (XLSX)

S2 Data Minimal data set for Figure 6: Loss of ZEB2 altered the expression of key factors required for proper ureteral SMC differentiation and development. (XLSX)

S3 Data Minimal data set for Supplementary Figure 5: Proliferation analysis in Zeb2 cKO mice. (XLSX)

## References

[1] Rasouly HM, Lu W. Lower urinary tract development and disease. Wiley Interdiscip Rev Syst Biol Med. 2013;5(3):307–42. doi: 10.1002/wsbm.1212 23408557 PMC3627353

[2] Bohnenpoll T, Kispert A. Ureter growth and differentiation. Semin Cell Dev Biol. 2014;36:21–30. doi: 10.1016/j.semcdb.2014.07.014 25087982

[3] Woolf AS, Davies JA. Cell biology of ureter development. J Am Soc Nephrol. 2013;24(1):19–25. doi: 10.1681/ASN.2012020127 23123402

[4] Bohnenpoll T, Feraric S, Nattkemper M, Weiss A-C, Rudat C, Meuser M, et al. Diversification of Cell Lineages in Ureter Development. J Am Soc Nephrol. 2017;28(6):1792–801. doi: 10.1681/ASN.2016080849 28028137 PMC5461796

[5] Airik R, Bussen M, Singh MK, Petry M, Kispert A. Tbx18 regulates the development of the ureteral mesenchyme. J Clin Invest. 2006;116(3):663–74. doi: 10.1172/JCI26027 16511601 PMC1386107

[6] Roth JA, Diamond DA. Prenatal hydronephrosis. Curr Opin Pediatr. 2001;13(2):138–41. doi: 10.1097/00008480-200104000-00009 11317055

[7] Woodward M, Frank D. Postnatal management of antenatal hydronephrosis. BJU Int. 2002;89(2):149–56. doi: 10.1046/j.1464-4096.2001.woodward.2578.x 11849184

[8] Mowat DR, Croaker GD, Cass DT, Kerr BA, Chaitow J, Adès LC, et al. Hirschsprung disease, microcephaly, mental retardation, and characteristic facial features: delineation of a new syndrome and identification of a locus at chromosome 2q22-q23. J Med Genet. 1998;35(8):617–23. doi: 10.1136/jmg.35.8.617 9719364 PMC1051383

[9] Cacheux V, Dastot-Le Moal F, Kääriäinen H, Bondurand N, Rintala R, Boissier B, et al. Loss-of-function mutations in SIP1 Smad interacting protein 1 result in a syndromic Hirschsprung disease. Hum Mol Genet. 2001;10(14):1503–10. doi: 10.1093/hmg/10.14.1503 11448942

[10] Wakamatsu N, Yamada Y, Yamada K, Ono T, Nomura N, Taniguchi H, et al. Mutations in SIP1, encoding Smad interacting protein-1, cause a form of Hirschsprung disease. Nat Genet. 2001;27(4):369–70. doi: 10.1038/86860 11279515

[11] Yamada K, Yamada Y, Nomura N, Miura K, Wakako R, Hayakawa C, et al. Nonsense and frameshift mutations in ZFHX1B, encoding Smad-interacting protein 1, cause a complex developmental disorder with a great variety of clinical features. Am J Hum Genet. 2001;69(6):1178–85. doi: 10.1086/324343 11592033 PMC1235530

[12] Adam MP, Feldman J, Mirzaa GM, Pagon RA, Wallace SE, Amemiya A. GeneReviews. 1993.

[13] Hossain WA, St Peter C, Lovell S, Rafi SK, Butler MG. ZEB2 Gene Pathogenic Variants Across Protein-Coding Regions and Impact on Clinical Manifestations: A Review. Int J Mol Sci. 2025;26(3):1307. doi: 10.3390/ijms26031307 39941075 PMC11818587

[14] St Peter C, Hossain WA, Lovell S, Rafi SK, Butler MG. Mowat-Wilson Syndrome: Case Report and Review of ZEB2 Gene Variant Types, Protein Defects and Molecular Interactions. Int J Mol Sci. 2024;25(5):2838. doi: 10.3390/ijms25052838 38474085 PMC10932183

[15] Garavelli L, Mainardi PC. Mowat-Wilson syndrome. Orphanet J Rare Dis. 2007;2:42. doi: 10.1186/1750-1172-2-42 17958891 PMC2174447

[16] Ivanovski I, Djuric O, Caraffi SG, Santodirocco D, Pollazzon M, Rosato S, et al. Phenotype and genotype of 87 patients with Mowat-Wilson syndrome and recommendations for care. Genet Med. 2018;20(9):965–75. doi: 10.1038/gim.2017.221 29300384

[17] Yamada Y, Nomura N, Yamada K, Matsuo M, Suzuki Y, Sameshima K, et al. The spectrum of ZEB2 mutations causing the Mowat-Wilson syndrome in Japanese populations. Am J Med Genet A. 2014;164A(8):1899–908. doi: 10.1002/ajmg.a.36551 24715670

[18] Garavelli L, Cerruti-Mainardi P, Virdis R, Pedori S, Pastore G, Godi M, et al. Genitourinary anomalies in Mowat-Wilson syndrome with deletion/mutation in the zinc finger homeo box 1B gene (ZFHX1B). Report of three Italian cases with hypospadias and review. Horm Res. 2005;63(4):187–92. doi: 10.1159/000085894 15908750

[19] Chng Z, Teo A, Pedersen RA, Vallier L. SIP1 mediates cell-fate decisions between neuroectoderm and mesendoderm in human pluripotent stem cells. Cell Stem Cell. 2010;6(1):59–70. doi: 10.1016/j.stem.2009.11.015 20074535

[20] McKinsey GL, Lindtner S, Trzcinski B, Visel A, Pennacchio LA, Huylebroeck D, et al. Dlx1&2-dependent expression of Zfhx1b (Sip1, Zeb2) regulates the fate switch between cortical and striatal interneurons. Neuron. 2013;77(1):83–98. doi: 10.1016/j.neuron.2012.11.035 23312518 PMC3547499

[21] Parthasarathy S, Srivatsa S, Nityanandam A, Tarabykin V. Ntf3 acts downstream of Sip1 in cortical postmitotic neurons to control progenitor cell fate through feedback signaling. Development. 2014;141(17):3324–30. doi: 10.1242/dev.114173 25085976

[22] Hegarty SV, Sullivan AM, O’Keeffe GW. Zeb2: A multifunctional regulator of nervous system development. Prog Neurobiol. 2015;132:81–95. doi: 10.1016/j.pneurobio.2015.07.001 26193487

[23] Hegarty SV, Wyatt SL, Howard L, Stappers E, Huylebroeck D, Sullivan AM, et al. Zeb2 is a negative regulator of midbrain dopaminergic axon growth and target innervation. Sci Rep. 2017;7(1):8568. doi: 10.1038/s41598-017-08900-3 28819210 PMC5561083

[24] Meyer SE. From EMT to HSC to AML: ZEB2 is a cell fate switch. Blood. 2017;129(4):400–1. doi: 10.1182/blood-2016-11-748186 28126955

[25] Menuchin-Lasowski Y, Oren-Giladi P, Xie Q, Ezra-Elia R, Ofri R, Peled-Hajaj S, et al. Sip1 regulates the generation of the inner nuclear layer retinal cell lineages in mammals. Development. 2016;143(15):2829–41. doi: 10.1242/dev.136101 27385012 PMC5004910

[26] Goossens S, Janzen V, Bartunkova S, Yokomizo T, Drogat B, Crisan M, et al. The EMT regulator Zeb2/Sip1 is essential for murine embryonic hematopoietic stem/progenitor cell differentiation and mobilization. Blood. 2011;117(21):5620–30. doi: 10.1182/blood-2010-08-300236 21355089

[27] Birkhoff JC, Huylebroeck D, Conidi A. ZEB2, the Mowat-Wilson Syndrome Transcription Factor: Confirmations, Novel Functions, and Continuing Surprises. Genes (Basel). 2021;12(7):1037. doi: 10.3390/genes12071037 34356053 PMC8304685

[28] Birkhoff JC, Brouwer RWW, Kolovos P, Korporaal AL, Bermejo-Santos A, Boltsis I, et al. Targeted chromatin conformation analysis identifies novel distal neural enhancers of ZEB2 in pluripotent stem cell differentiation. Hum Mol Genet. 2020;29(15):2535–50. doi: 10.1093/hmg/ddaa141 32628253 PMC7471508

[29] Rasouly HM, Kumar S, Chan S, Pisarek-Horowitz A, Sharma R, Xi QJ, et al. Loss of Zeb2 in mesenchyme-derived nephrons causes primary glomerulocystic disease. Kidney Int. 2016;90(6):1262–73. doi: 10.1016/j.kint.2016.06.037 27591083 PMC5123919

[30] Kumar S, Fan X, Rasouly HM, Sharma R, Salant DJ, Lu W. ZEB2 controls kidney stromal progenitor differentiation and inhibits abnormal myofibroblast expansion and kidney fibrosis. JCI Insight. 2023;8(1):e158418. doi: 10.1172/jci.insight.158418 36445780 PMC9870089

[31] Higashi Y, Maruhashi M, Nelles L, Van de Putte T, Verschueren K, Miyoshi T, et al. Generation of the floxed allele of the SIP1 (Smad-interacting protein 1) gene for Cre-mediated conditional knockout in the mouse. Genesis. 2002;32(2):82–4. doi: 10.1002/gene.10048 11857784

[32] Cai C-L, Martin JC, Sun Y, Cui L, Wang L, Ouyang K, et al. A myocardial lineage derives from Tbx18 epicardial cells. Nature. 2008;454(7200):104–8. doi: 10.1038/nature06969 18480752 PMC5540369

[33] Trowe M-O, Airik R, Weiss A-C, Farin HF, Foik AB, Bettenhausen E, et al. Canonical Wnt signaling regulates smooth muscle precursor development in the mouse ureter. Development. 2012;139(17):3099–108. doi: 10.1242/dev.077388 22833126

[34] Southgate J, Harnden P, Trejdosiewicz LK. Cytokeratin expression patterns in normal and malignant urothelium: a review of the biological and diagnostic implications. Histol Histopathol. 1999;14(2):657–64. doi: 10.14670/HH-14.657 10212826

[35] Charney RM, Prasad MS, Juan-Sing C, Patel LJ, Hernandez JC, Wu J, et al. Mowat-Wilson syndrome factor ZEB2 controls early formation of human neural crest through BMP signaling modulation. Stem Cell Reports. 2023;18(11):2254–67. doi: 10.1016/j.stemcr.2023.10.002 37890485 PMC10679662

[36] Bohnenpoll T, Weiss A-C, Labuhn M, Lüdtke TH, Trowe M-O, Kispert A. Retinoic acid signaling maintains epithelial and mesenchymal progenitors in the developing mouse ureter. Sci Rep. 2017;7(1):14803. doi: 10.1038/s41598-017-14790-2 29093497 PMC5665985

[37] Bohnenpoll T, Wittern AB, Mamo TM, Weiss A-C, Rudat C, Kleppa M-J, et al. A SHH-FOXF1-BMP4 signaling axis regulating growth and differentiation of epithelial and mesenchymal tissues in ureter development. PLoS Genet. 2017;13(8):e1006951. doi: 10.1371/journal.pgen.1006951 28797033 PMC5567910

[38] Weiss A-C, Blank E, Bohnenpoll T, Kleppa M-J, Rivera-Reyes R, Taketo MM, et al. Permissive ureter specification by TBX18-mediated repression of metanephric gene expression. Development. 2023;150(6):dev201048. doi: 10.1242/dev.201048 36960826

[39] Airik R, Trowe M-O, Foik A, Farin HF, Petry M, Schuster-Gossler K, et al. Hydroureternephrosis due to loss of Sox9-regulated smooth muscle cell differentiation of the ureteric mesenchyme. Hum Mol Genet. 2010;19(24):4918–29. doi: 10.1093/hmg/ddq426 20881014

[40] Kispert A. Ureter development and associated congenital anomalies. Nat Rev Nephrol. 2025;21(6):366–82. doi: 10.1038/s41581-025-00951-4 40164775

[41] Tripathi P, Wang Y, Casey AM, Chen F. Absence of canonical Smad signaling in ureteral and bladder mesenchyme causes ureteropelvic junction obstruction. J Am Soc Nephrol. 2012;23(4):618–28. doi: 10.1681/ASN.2011060566 22282597 PMC3312498

[42] Yan J, Zhang L, Xu J, Sultana N, Hu J, Cai X, et al. Smad4 regulates ureteral smooth muscle cell differentiation during mouse embryogenesis. PLoS One. 2014;9(8):e104503. doi: 10.1371/journal.pone.0104503 25127126 PMC4134214

[43] Nie X, Sun J, Gordon RE, Cai C-L, Xu P-X. SIX1 acts synergistically with TBX18 in mediating ureteral smooth muscle formation. Development. 2010;137(5):755–65. doi: 10.1242/dev.045757 20110314 PMC2827686

[44] Cain JE, Islam E, Haxho F, Blake J, Rosenblum ND. GLI3 repressor controls functional development of the mouse ureter. J Clin Invest. 2011;121(3):1199–206. doi: 10.1172/JCI45523 21339645 PMC3049374

[45] Miyazaki Y, Oshima K, Fogo A, Ichikawa I. Evidence that bone morphogenetic protein 4 has multiple biological functions during kidney and urinary tract development. Kidney Int. 2003;63(3):835–44. doi: 10.1046/j.1523-1755.2003.00834.x 12631064

[46] Brenner-Anantharam A, Cebrian C, Guillaume R, Hurtado R, Sun T-T, Herzlinger D. Tailbud-derived mesenchyme promotes urinary tract segmentation via BMP4 signaling. Development. 2007;134(10):1967–75. doi: 10.1242/dev.004234 17442697

[47] Quintes S, Brinkmann BG, Ebert M, Fröb F, Kungl T, Arlt FA, et al. Zeb2 is essential for Schwann cell differentiation, myelination and nerve repair. Nat Neurosci. 2016;19(8):1050–9. doi: 10.1038/nn.4321 27294512 PMC4964942

[48] Wu LMN, Wang J, Conidi A, Zhao C, Wang H, Ford Z, et al. Zeb2 recruits HDAC-NuRD to inhibit Notch and controls Schwann cell differentiation and remyelination. Nat Neurosci. 2016;19(8):1060–72. doi: 10.1038/nn.4322 27294509 PMC4961522

[49] Cheng P, Wirka RC, Shoa Clarke L, Zhao Q, Kundu R, Nguyen T, et al. ZEB2 Shapes the Epigenetic Landscape of Atherosclerosis. Circulation. 2022;145(6):469–85. doi: 10.1161/CIRCULATIONAHA.121.057789 34990206 PMC8896308

[50] Epifanova E, Babaev A, Newman AG, Tarabykin V. Role of Zeb2/Sip1 in neuronal development. Brain Res. 2019;1705:24–31. doi: 10.1016/j.brainres.2018.09.034 30266271

[51] Nishizaki Y, Takagi T, Matsui F, Higashi Y. SIP1 expression patterns in brain investigated by generating a SIP1-EGFP reporter knock-in mouse. Genesis. 2014;52(1):56–67. doi: 10.1002/dvg.22726 24243579

