# Supplementary figures and images for: Transcription factor ZEB2 is essential for ureteral smooth muscle cell differentiation
Source: PLoS Genet. 2026 Jan 23;22(1):e1012028. doi: 10.1371/journal.pgen.1012028 (PMC12900438; doi:10.1371/journal.pgen.1012028)

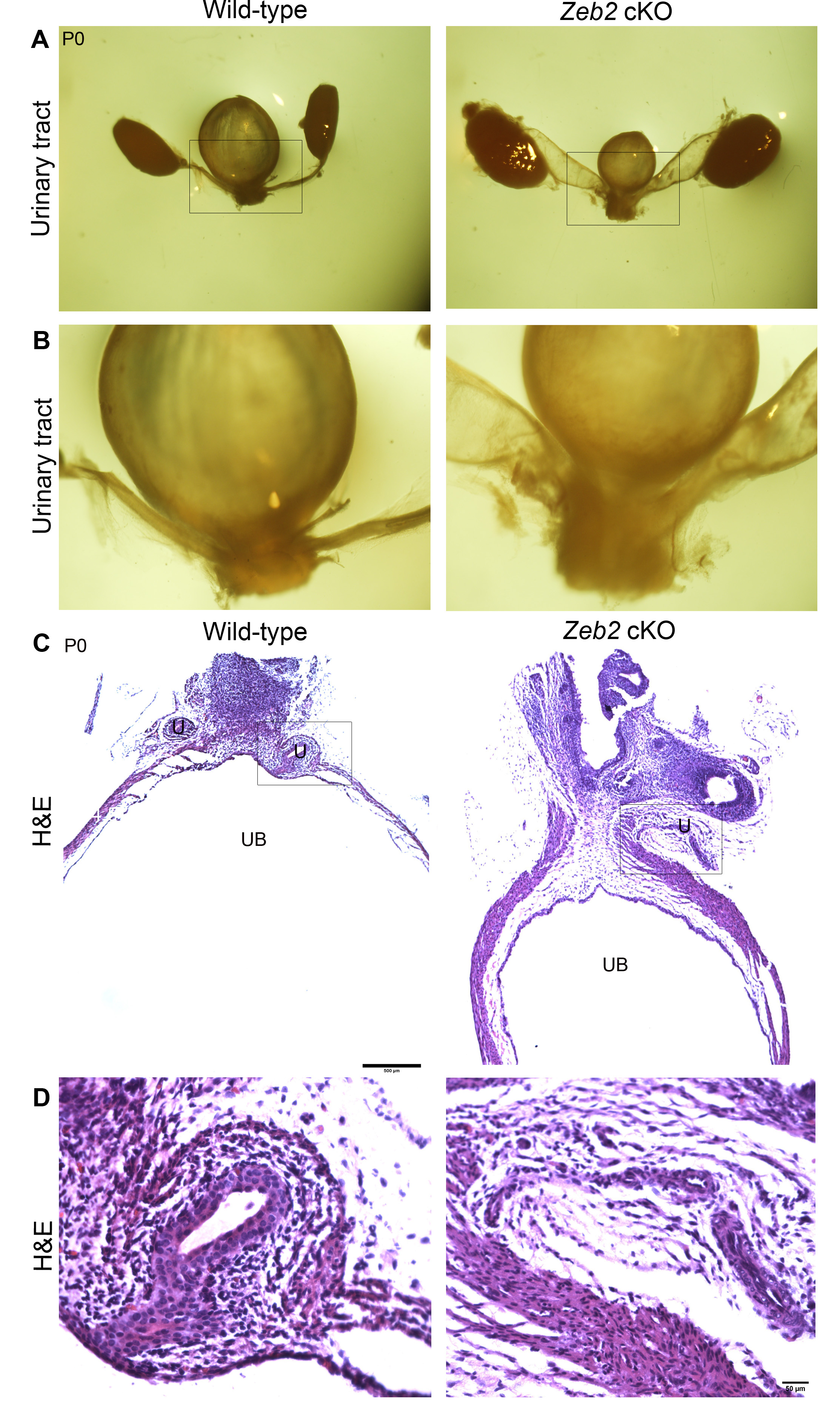

Supplement: S1 Fig — (A) Whole urinary tract system of P0 Zeb2 cKO mice and wild-type controls. (B) Highlighted area in panel A. (C) H&E staining of UVJ in P0 Zeb2 cKO mice and wild-type controls. Zeb2 cKO mice. (D) Highlighted area in panel C. Zeb2 cKO mice had partial obstruction of the UVJ as compared with wild-type control mice. Number of animals analyzed: n = 3 per group. U = ureter, UB = Urinary bladder. (JPG) [file pgen.1012028.s001.jpg]

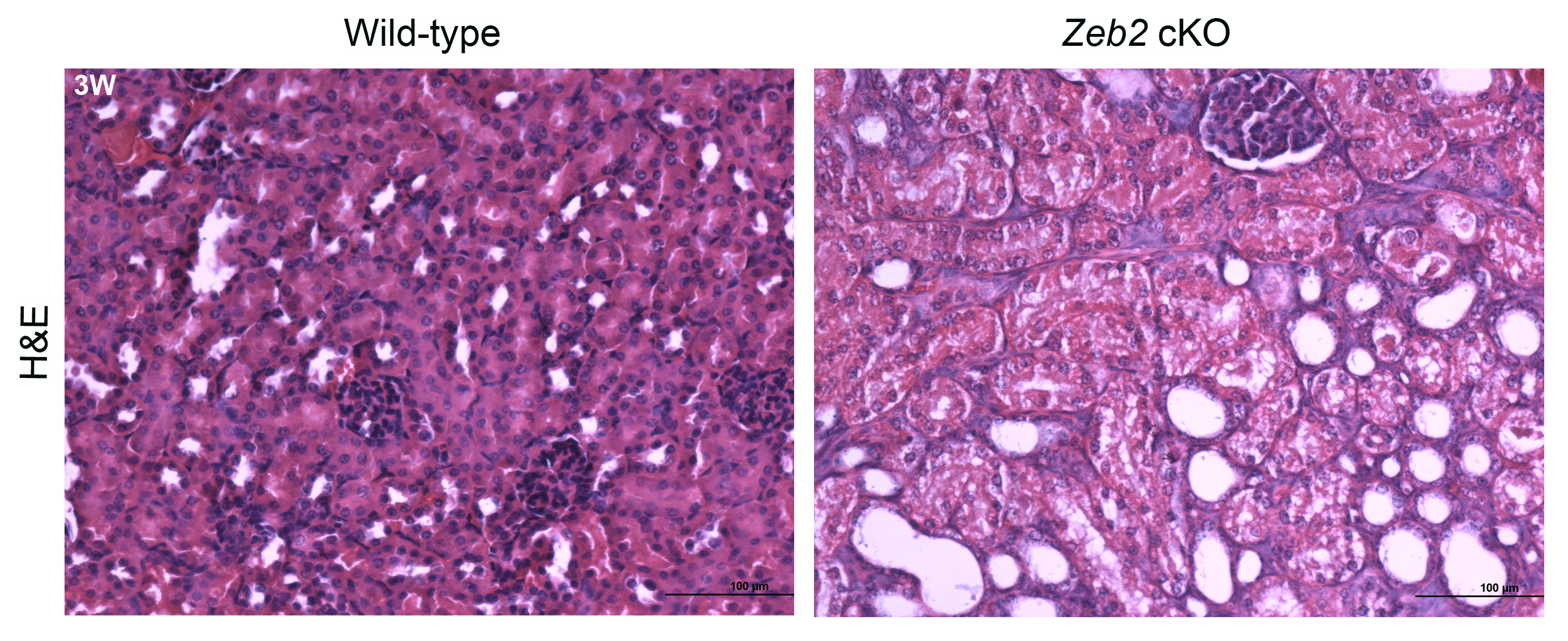

Supplement: S2 Fig — Zeb2 cKO mice had significantly dilated tubules due to hydronephrosis. Number of animals analyzed: n = 3 per group. (JPG) [file pgen.1012028.s002.jpg]

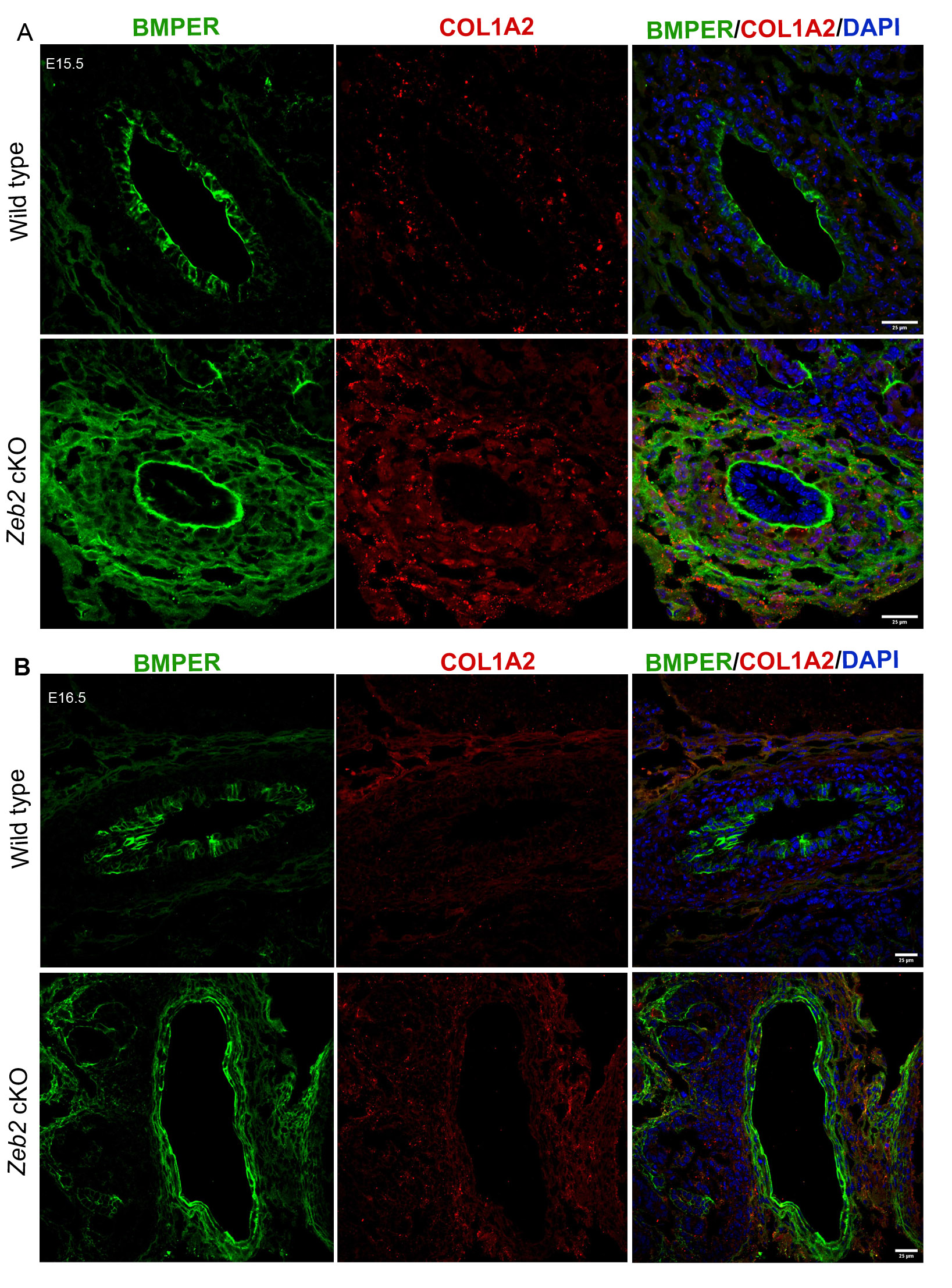

Supplement: S3 Fig — (A) Immunofluorescent staining of BMP-binding endothelial regulator (BMPER, green) and collagen 1a2 (COL1A2, red) at the proximal ureter sections at E15.5 in the Zeb2 cKO mice and wild-type controls. (B) Immunofluorescent staining of BMP-binding endothelial regulator (BMPER, green) and collagen 1a2 (COL1A2, red) at the proximal ureter sections at E16.5 in the Zeb2 cKO mice and wild-type controls. Scale bars: 25 µm (A-B). Number of animals analyzed: n = 3 per group. (JPG) [file pgen.1012028.s003.jpg]

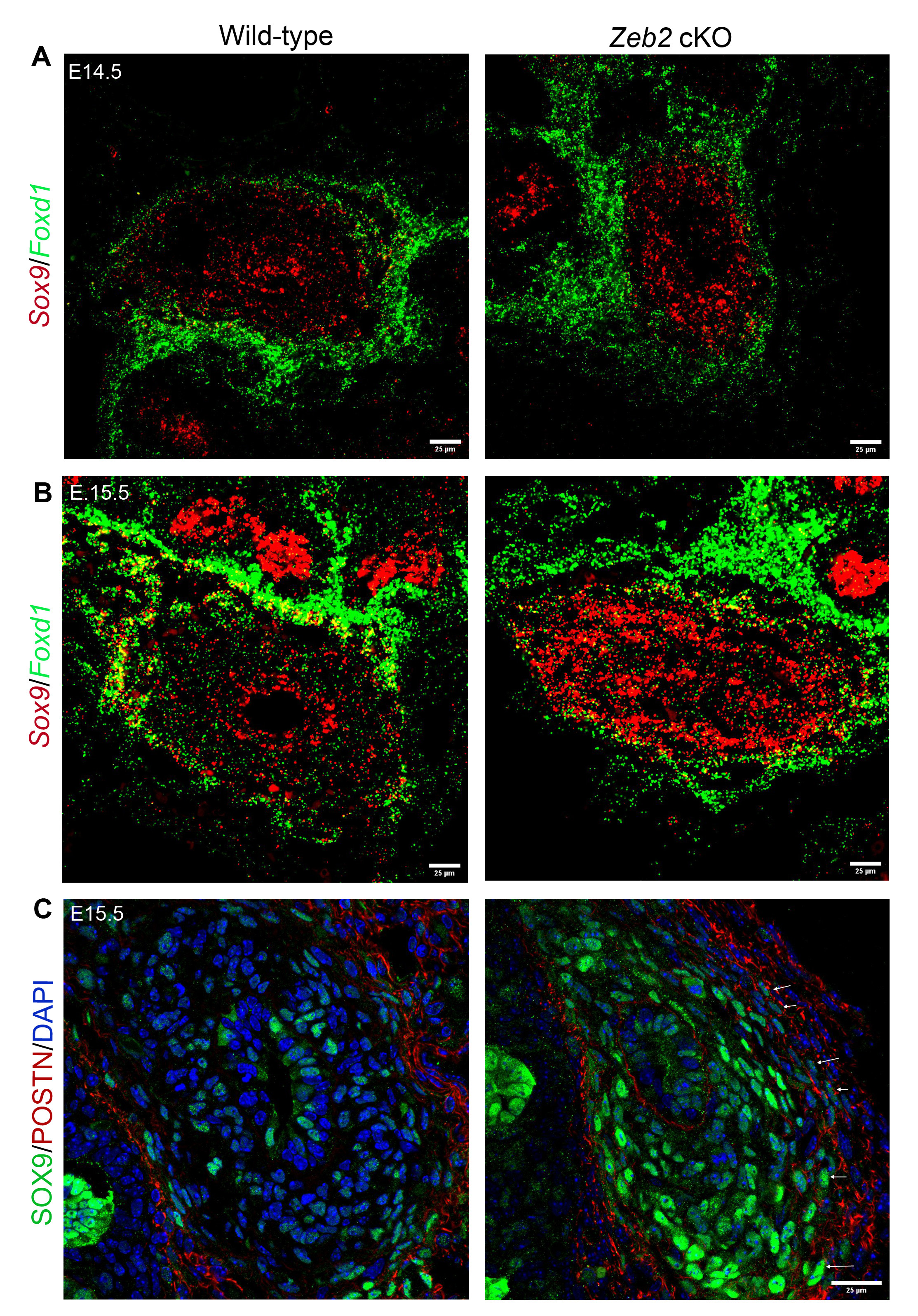

Supplement: S4 Fig — (A) RNAscope of Foxd1 (green) and Sox9 (red) at the proximal ureter sections at E14.5 in the Zeb2 cKO mice and wild-type controls. (B) RNAscope of Foxd1 (green) and Sox9 (red) at the proximal ureter sections at E15.5 in the Zeb2 cKO mice and wild-type controls. (C) Immunofluorescent staining of SOX9 (green) and POSTN (red) at the proximal ureter sections at E15.5 in the Zeb2 cKO mice and wild-type controls. The expanded SOX9 expression was marked with arrows in panel C. Scale bars: 25 µm (A-C). Number of animals analyzed: n = 3 per group. (JPG) [file pgen.1012028.s004.jpg]

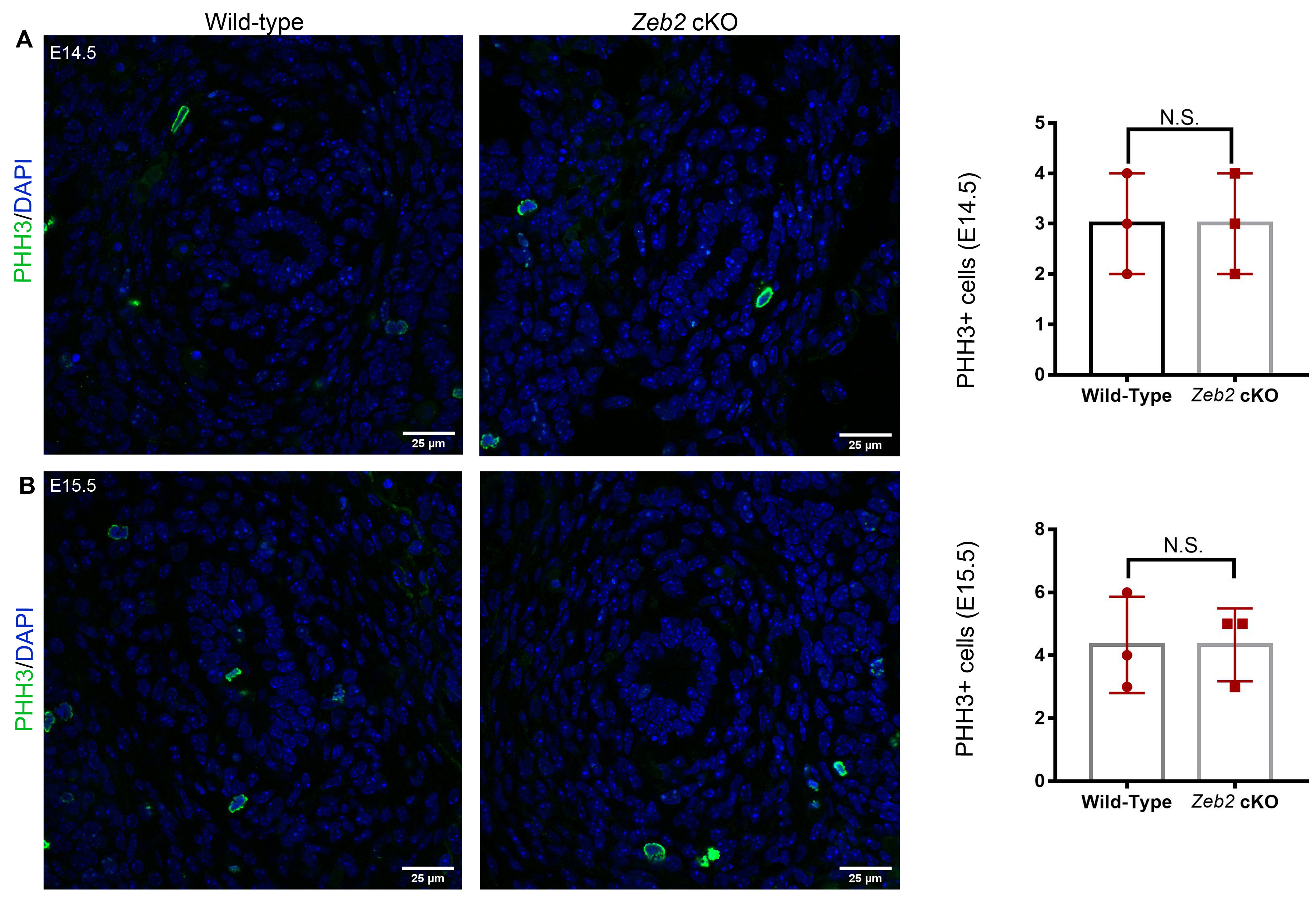

Supplement: S5 Fig — (A) Immunofluorescent staining of PHH3 at the proximal ureter sections at E14.5 in the Zeb2 cKO mice and wild-type controls. (B) Immunofluorescent staining of PHH3 at the proximal ureter sections at E15.5 in the Zeb2 cKO mice and wild-type controls. Scale bars: 25 µm (A-B). Number of animals analyzed: n = 3 per group. Values are expressed as mean ± SEM. Student’s t-test is used for statistical significance. N.S. = not significant. (JPG) [file pgen.1012028.s005.jpg]

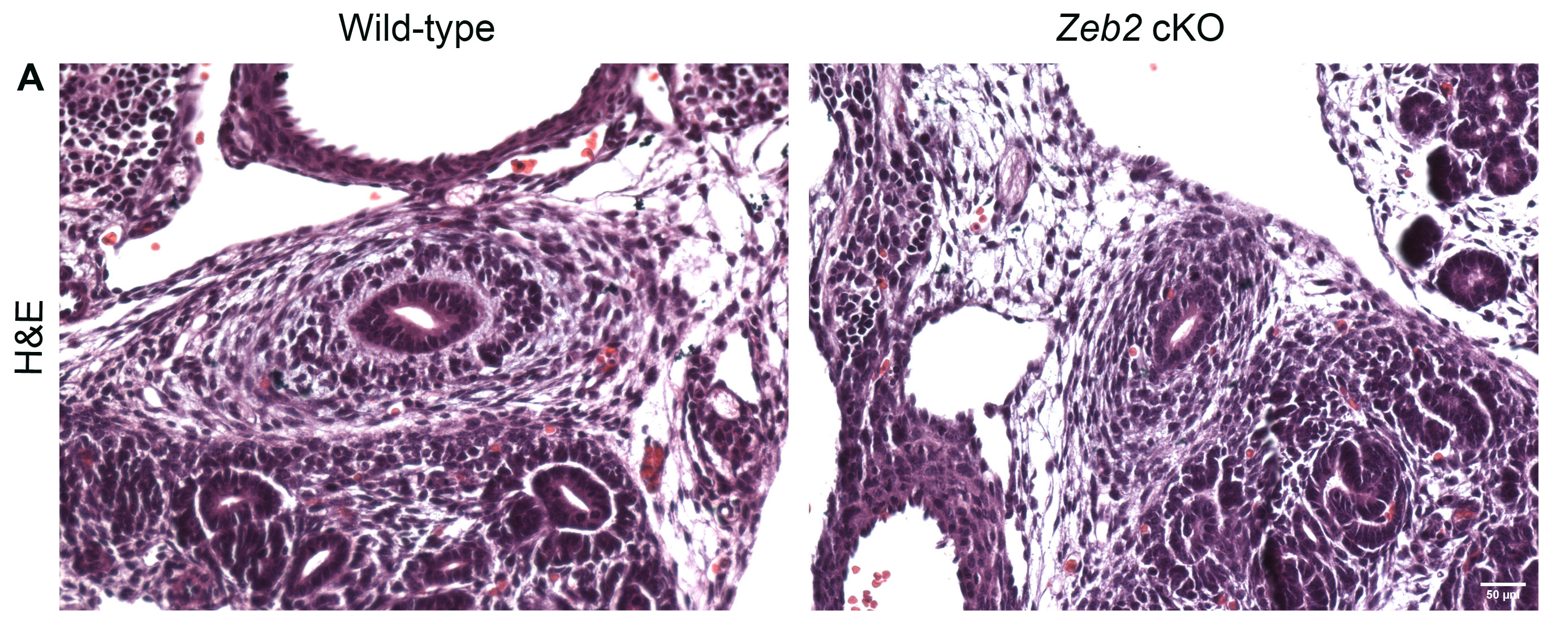

Supplement: S6 Fig — (A) H&E staining showing normal condensation of ureteral mesenchymal cells in the wild-type mice at E14.5, but it was compacted and disorganized in Zeb2 cKO mice. Scale bars: 50 µm. Number of animals analyzed: n = 3 per group. (JPG) [file pgen.1012028.s006.jpg]
